# Microbial Electrolysis Cells Based on a Bacterial Anode Encapsulated with a Dialysis Bag Including Graphite Particles

**DOI:** 10.3390/microorganisms12071486

**Published:** 2024-07-20

**Authors:** Irina Amar Dubrovin, Lea Ouaknin Hirsch, Abhishiktha Chiliveru, Avinash Jukanti, Shmuel Rozenfeld, Alex Schechter, Rivka Cahan

**Affiliations:** 1 Department of Chemical Engineering, Ariel University, Ariel 40700, Israel; irinadubrovin@gmail.com (I.A.D.); lea.ouaknin@gmail.com (L.O.H.); abhishikthachilivery@gmail.com (A.C.); javinash1007@gmail.com (A.J.); shmulik2009@gmail.com (S.R.); 2 Department of Chemistry, Ariel University, Ariel 40700, Israel; salex@ariel.ac.il

**Keywords:** microbial electrolysis cell, bacterial anode, anode encapsulation, graphite nanoparticles

## Abstract

One of the main barriers to MEC applicability is the bacterial anode. Usually, the bacterial anode contains non-exoelectrogenic bacteria that act as a physical barrier by settling on the anode surface and displacing the exoelectrogenic microorganisms. Those non-exoelectrogens can also compete with exoelectrogenic microorganisms for nutrients and reduce hydrogen production. In this study, the bacterial anode was encapsulated by a dialysis bag including suspended graphite particles to improve current transfer from the bacteria to the anode material. An anode encapsulated in a dialysis bag without graphite particles, and a bare anode, were used as controls. The MEC with the graphite-dialysis-bag anode was fed with artificial wastewater, leading to a current density, hydrogen production rate, and areal capacitance of 2.73 A·m^−2^, 134.13 F·m^−2^, and 7.6 × 10^−2^ m^3^·m^−3^·d^−1^, respectively. These were highest when compared to the MECs based on the dialysis-bag anode and bare anode (1.73 and 0.33 A·m^−2^, 82.50 and 13.75 F·m^−2^, 4.2 × 10^−2^ and 5.2 × 10^−3^ m^3^·m^−3^·d^−1^, respectively). The electrochemical impedance spectroscopy of the modified graphite-dialysis-bag anode showed the lowest charge transfer resistance of 35 Ω. The COD removal results on the 25th day were higher when the MEC based on the graphite-dialysis-bag anode was fed with *Geobacter* medium (53%) than when it was fed with artificial wastewater (40%). The coulombic efficiency of the MEC based on the graphite-dialysis-bag anode was 12% when was fed with *Geobacter* medium and 15% when was fed with artificial wastewater.

## 1. Introduction

Our environment faces severe pollution problems due to increases in the world’s population and global energy demand. The possible solutions may include switching to eco-friendly and sustainable (renewable) energy sources. Many scientific efforts are focusing on waste-to-energy conversion technologies [1,2]; for example, the conversion of feedstock into hydrogen gas, electricity, and bioethanol [3,4].

Hydrogen can be produced either on-site at its point of use or centrally with subsequent distribution. Additionally, it can be derived from diverse sources such as water, natural gas, and biomass [5,6]. Hydrogen is an efficient fuel, delivering a significant amount of energy when compared to diesel or gasoline. It yields three times the energy content per unit of weight compared to gasoline or methane (120–142 MJ·kg^−1^ for H_2_ vs. 50 MJ·kg^−1^ for CH_4_ or 44 MJ·kg^−1^ for gasoline) [7,8].

Microbial electrochemical technologies (METs), such as microbial fuel cells (MFCs) and microbial electrolysis cells (MECs), represent innovative approaches to energy recovery [9]. METs are composed of anode and cathode compartments. The bacterial anode oxidizes organic matter into electrons and protons. The electrons move to the cathode by the bacterial anode through a conductive wire, while the protons move according to gradient concentration. In MFCs, there is electricity generation under external resistance and aerobic conditions in the cathode compartment [10]. In MECs, there is a production of hydrogen under low voltage and anaerobic conditions in the cathode compartment [11].

The efficiency of hydrogen production in MECs is highly dependent on the performance of the anodic biofilm [12,13]. Various strategies have been explored to enhance biofilm growth, including the use of different electrode materials like carbon or graphite, in various forms such as felt, brushes, cloths, tubes, or plates. The preference for carbon as the anode material is due to its biocompatibility, which enables electrochemically active biofilm formation, and its cost-effectiveness [14,15]. Graphite, a highly conductive material, is an excellent electron-transfer mediator, facilitating the movement of electrons from the bacterial cells to the anode surface [16]. Moreover, graphite particles can also promote biofilm formation on the anode surface, providing a larger surface area for bacterial attachment [17]. Hirsch et al. (2023) studied the immobilization of the bacterial anode with kaolin (a clay mineral) and activated carbon to improve bacterial attachment and electron transfer to the anode material. The authors reported a higher power density of 56% compared to a control bacterial anode, and a coulombic efficiency of 16% [18]. Cheng and Logan (2007) constructed an MFC with an anode chamber filled with graphite granules. The results showed an HER of 1.5 m^3^·m^−3^·d^−1^ and a hydrogen yield of 3.95 mol·mol^−1^ at an applied voltage of 0.8 V [19]. Yong et al. (2013) used graphite-alginate granules to immobilize *Shewanella oneidensis* MR-1 in an anodic chamber of an MFC; the immobilized cells showed a resistance and charge transfer resistance of 19 Ω and 1600 Ω, respectively [20].

Some studies have encapsulated the bioanode in an attempt to separate exoelectrogenic bacteria from non-exoelectrogenic bacteria. In our previous study, we showed a novel encapsulation technology based on a small bioreactor platform (SBP). The 3D capsule physically separates the biofilm on the anode material from the natural microorganisms in the wastewater, while enabling the diffusion of nutrients through the capsule membrane. When the MECs were fed with artificial wastewater, the MEC-SBP produced 1.70 ± 0.22 A·m^−2^ (at 0.6 V), twice that of the control MEC which was based on a bare anode. The HERs were 0.017 and 0.005 m^3^·m^−3^·day^−1^, respectively. The COD consumption rate for both was about the same at 650 ± 70 mg·L^−1^ [21]. Rozenfeld et al. (2021) focused on a semi-single-chamber MEC based on a carbon cloth anode combined with stainless steel and encapsulated in a dialysis bag. A dialysis bag is made of a semi-permeable membrane allowing the exchange of ions and small molecules. Encapsulation of the anode in a dialysis bag prevents direct contact between the anodic and cathodic environments. This configuration also provides a controlled environment for the anodic exoelectrogenic bacteria, protecting them from the invasion of non-exoelectrogenic bacteria and ensuring their optimal electroactivity performance. The current density and HER obtained in the MEC based on the dialysis-bag anodes were 10.39 A·m^−2^ and 0.160 ± 0.009 m^3^·m^−2^·d^−1^, respectively, while the control MEC utilizing a bare anode led to only 5.50 A·m^−2^ and 0.122 ± 0.004 m^3^·m^−2^·d^−1^, respectively [22].

In this study, the bacterial anode was encapsulated in a dialysis bag including *G. sulfurreducens* to separate the exoelectrogenic bacteria from non-electrogenic bacteria in wastewater. Graphite particles were used to increase the electron transfer from the bacteria to the anode material. The MECs were fed with *Geobacter* medium or artificial wastewater. The electrochemical activity, COD removal, viability of the anodic biofilm and the planktonic bacteria, and the relative bacterial anode distribution of the MECs based on the encapsulated anode in a dialysis bag, with and without graphite particles, were then compared to an MEC based on a bare anode.

## 2. Materials and Methods

### 2.1. Electrode Preparation

Carbon cloth material (Plain Carbon Cloth 1071; FuelCellsEtc, Bryan, TX, USA) was used as the bacterial anode. To increase the anode surface hydrophilicity, the carbon cloth was cut into 2.5 × 2.5 cm sheets, which were inserted into a cold low-pressure plasma device (Harrick PDC-32G2-, New York, NY, USA) and treated with nitrogen gas under a pressure of 2 torr for 2 min. To preserve the carbon cloth’s hydrophilic nature, it was placed in a chamber containing double-distilled water [23].

The modified anode was constructed as follows: a plasma-treated carbon cloth was encapsulated in a dialysis bag containing extra-pure graphite powder (<50 µm) (0.25 g) (Merck, Darmstadt, Germany) and designated as the graphite-dialysis-bag anode. A carbon cloth anode that was encapsulated without graphite powder (designated as the dialysis-bag anode), and a bare carbon cloth anode (designated as the bare anode) were used as controls. Carbon cloth material coated with 0.5 mg·cm^−^^2^ Pt 60% (Cloth GDE; FuelCellsEtc, Bryan, TX, USA) with a geometric area of 6.25 cm^2^ was used as a cathode. The reference Ag/AgCl electrode (3.0 M KCl, +199 mV vs. SHE; ALS Co., Ltd., Osaka, Japan) was used for electrochemical tests. Four pure titanium wires (Gr1 grade 0.3 mm, 25 cm) isolated with synthetic polymer (Plastidip, Yokneam Illit, Israel) connected the electrodes to the potentiostat.

### 2.2. Dialysis Bag Preparation

Dialysis tubing (SpectraPor^®^ 7 RC dialysis membrane, 50 kDa; Gardena, CA, USA) was cut to a desired length (20 cm for each bag), and immersed into 1 L of 2% sodium bicarbonate and 1 mM EDTA, followed by boiling for 10 min. The tubing was rinsed thoroughly with double-distilled water (ddH_2_O) and boiled for 10 min in ddH_2_O. Then, the dialysis tubing was inserted in 50% ethanol: 1 mM EDTA and kept until used.

### 2.3. Preparation of Artificial Wastewater

**Figure 1:**
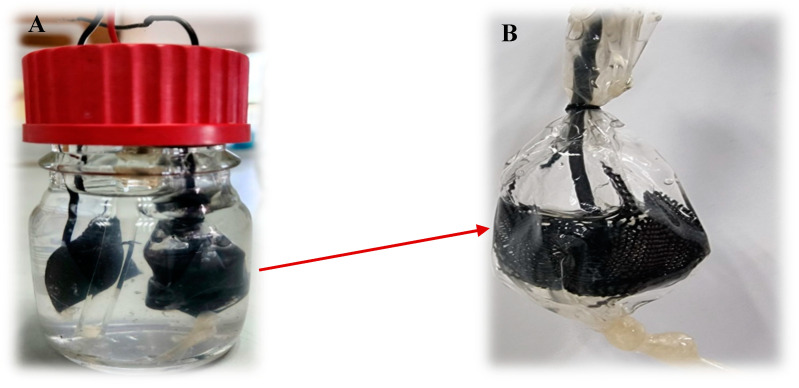
MEC based on graphite dialysis bag anode (**A**). Dialysis bag anode (**B**).

*Escherichia coli* (DSMZ 6899) and *Enterobacter cloacae* (DSMZ 30054) were grown to the logarithmic phase in brain-heart broth (BHB) (Himedia, Maharashtra, India) in 50 mL tubes for about 3 h. Each culture was diluted to 0.10 ± 0.05 OD 660 nm. The artificial wastewater included 1 mL of each strain in BHB (total of 2 mL), along with 10 mL yeast extract (YE) (Himedia, India), 70 mL *Geobacter* medium, and 18 mL phosphate buffer (PB), pH 6.8 (Figure 1).

### 2.4. MEC System Setup

The single-chamber MEC system consisted of a 100 mL glass bottle with a septum cap made of silicone/PTFE. All the MEC components were sterilized, except for the anode (to preserve the effect of plasma treatment) and the cathode (to avoid damage to the Pt coating). Three electrodes: cathode, reference electrode, and anode (dialysis-bag anode, graphite-dialysis-bag anode, or bare anode) were inserted through the septum cap and connected to a potentiostat (MultiEmStat3+, Palmsens; CL Houten, Vleugelboot, Houten, The Netherlands). The MECs were filled with *Geobacter* medium (N’ 826, DSMZ, Braunschweig, Germany) including sodium acetate (2 g·L^−1^) as the carbon source (80 mL) and 20 mL of phosphate buffer (PB) (100 mM, pH 6.8) (designated as *Geobacter* medium) or 100 mL artificial wastewater. The MECs were placed on a magnetic stirrer platform at room temperature (22 °C) and stirred (350 rpm) for 28 days, at a constant potential of 0.3 V vs. Ag/AgCl (3.0 M KCl).

### 2.5. Inoculation of G. sulfurreducens into the MEC

A pure culture of *G. sulfurreducens* (DSMZ 12127) was grown for 10 days and concentrated to 0.30 ± 0.05 OD. A bacterial suspension of 3 mL [21] was injected directly into the dialysis bag. Then, the dialysis bag was bound with a thread. The MEC with the bare anode was inoculated with the same volume and bacterial density into the whole liquid volume (100 mL).

### 2.6. Electrochemical Measurements

The MEC was connected to a MultiEmStat3+ potentiostat. The anode’s bio-electrochemical activity was examined by cyclic voltammetry (CV) at an applied potential range of −0.8 to 0.8 V. Linear sweep voltammetry (LSV) was performed in two different modes. The anode’s electroactive oxidation currents were generated in a three-electrode configuration at an applied potential range of −0.5 to 0.8 V vs. Ag/AgCl (3.0 M KCl). For the cathode’s electroactive reduction currents, the investigation was performed in a two-electrode configuration at an applied potential range of 0 to −0.8 V vs. Ag/AgCl (3.0 M KCl). All the CV and LSV analyses were carried out at a scan rate of 5 mV·s^−1^ to allow steady-state polarization conditions.

Electrochemical impedance spectroscopy (EIS) measurements of the microbial anodes were conducted to evaluate various resistances associated with the charge transfer (Rct) accumulation in the studied electrode processes. The microbial anode was controlled by a potentiostat (Ivium N-Stat; Eindhoven, The Netherlands) in a three-electrode configuration under an applied bias potential of 0 V vs. Ag/AgCl 3 M KCl (0.226 V). AC voltage perturbation amplitude of 10 mV in the frequency range of 10^5^–10^−1^ Hz frequency was applied around the bias potential. The resulting EIS spectra were analyzed using ZView software (ZSimpWin 3.21). The electrochemical results are based on 3 replicates.

The calculations of hydrogen production rate (HER), coulombic hydrogen recovery (coulombic efficiency) (${CE}_{H_{2}}$) and areal capacitance (*C_A_*) under applied constant potentials were performed according to Equations (1)–(4) [9,24].

(1) $$U_{H_{2}}=\frac{I\times R\times T}{z\times F\times P}$$

where $U_{H_{2}}$—hydrogen production volume (m^3^), *P*—gas pressure (atm), *z*—valence of an element, *R*—the gas constant (0.0820577 L·atm·mol^−1^·K^−1^), *T*—gas temperature (K), *I*—current (A), and *F*—Faraday’s constant (96,485 C·mol^−1^).

(2) $$HER=\frac{U_{H_{2}}}{V_{R}\times t}$$

where HER—hydrogen production rate, $U_{H_{2}}—$hydrogen production volume (m^3^, calculated from Equation (1)), $V_{R}$—reactor volume, and *t*—time in seconds normalized to 1 day.

(3) $${C_{E}}_{H_{2}}=\frac{M_{W_{s}}\times \int_{t=0}^{t=s}Idt\times 100\%}{2F\times b_{H2/s}\times V_{r}\times ∆COD}$$

where ${CE}_{H_{2}}$—coulombic hydrogen recovery (coulombic efficiency), $M_{W}$—molar mass of substrate (g·mol^−1^), *I*—current (A), *F*—Faraday’s constant (96,485 C·mol^−1^), $b_{H2/s}$—stoichiometric number of moles of hydrogen produced per mole of substrate (mole H_2_·mole substrate^−1^), and $V_{r}$—reactor volume normalized to cubic meters (m^3^).

(4) $$C_{A}=\frac{A_{CV}}{2\times k\times (V_{1}-V_{2})}$$

where $C_{A}$—areal capacitance (F·m^−2^), *A_CV_*—CV curve absolute area normalized to the area of active materials (A·V·m^−2^), *k*—scan rate (V·s^−1^), and $\left(V_{1}-V_{2}\right)$—potential window (V).

### 2.7. Chemical Oxygen Demand Assay

A high-range (0–15,000 mg·L^−1^) kit (Lovibond™ COD tube tests; HR, Amesbury, UK) was used to examine chemical oxygen demand (COD), which is the amount of dissolved oxygen in water required for oxidizing chemical organic components. Each sample (0.2 mL) that was taken from the MEC liquid was filtered (0.22 um), diluted, and merged into the kit tube containing potassium dichromate, sulphuric acid, and metal salts. The sample (3 replicates of each sample) was gently mixed and incubated in a COD reactor (DBR-001; MRC, Holon, Israel) for 2 h at 150 °C. A spectrophotometer (430–610 nm range) (Photometer-system MD 100; Lovibond™, Berlin, Germany) was used to evaluate the absorbance of the digested solution.

### 2.8. Measurement of the Bacterial Viability of the Anode Biofilm and the Suspended Bacteria

At the end of the MEC operation, the viability of the biofilm on the anodes, and of the suspended bacteria in the dialysis bag or in the whole volume of the MEC, was examined using the 3-(4,5-Dimethylthiazol-2-yl)-2,5-diphenyltetrazolium bromide reagent (MTT; Merck, Darmstadt, Germany). The MTT assay is based on the reduction of the tetrazolium reagent by bacterial hydrogenase activity. The intensity of the purple color of the tetrazolium reagent was measured using a GENESYS 10S UV-Visible spectrophotometer (Thermo Scientific, Waltham, MA, USA).

For measuring the bacterial anode biofilm, the anode (3.125 cm^2^), with its attached biofilm, was rinsed three times with PBS to remove planktonic bacteria. Subsequently, the anode was placed in a 15 mL tube with 5 mL of MTT solution (500 ppm) and incubated in the dark at 30 °C for 2 h. Then, the MTT solution was removed, and 5 mL of dimethyl sulfoxide–ethanol solution (1:1 ratio) was added for 40 min to facilitate the color development of the reduced MTT. The absorbance of the resulting purple solution was then measured at 540 nm [21].

A sample of one mL of suspended bacteria in the dialysis bag, or in the whole volume of the MEC based on the bare anode, was collected and decanted into an Eppendorf tube to measure the bacterial viability. The sample was centrifuged (10,000 rpm, 4 min), and the supernatant was replaced with 5 mL MTT solution (500 ppm), followed by incubation in the dark at 30 °C for 2 h. The sample was again centrifuged (10,000 rpm, 4 min), and the supernatant was replaced with 5 mL dimethyl sulfoxide–ethanol solution (1:1 ratio) for another 40 min. The absorbance of the resulting purple solution was then measured at 540 nm. The suspended bacteria or the biofilm viability results are based on 3 replicates.

### 2.9. Relative Bacterial Distribution Analysis of the Bioanode Community

The relative microbial distribution analysis was performed on the MECs based on a dialysis-bag anode, graphite-dialysis-bag anode, and bare anode by HyLabs Ltd. (Rehovot, Israel). The DNeasy Powersoil kit (Qiagen, Hilden, Germany) was used to extract the DNA. A 16S library preparation for sequencing on Illumina (San Diego, CA, USA) was performed using a 2-step PCR protocol. Sequencing was done on the Illumina Miseq, using a v2-500 cycles kit to generate 2 × 250 paired-end readings. Demultiplexing was performed on Basespace (the Illumina cloud) to generate FASTQ files for each sample. The data was further analyzed using CLC-bio (Aarhus, Denmark) to generate OTU and Abundance tables [21].

## 3. Results and Discussion

### 3.1. Voltammetric Measurements

**Figure 2:**
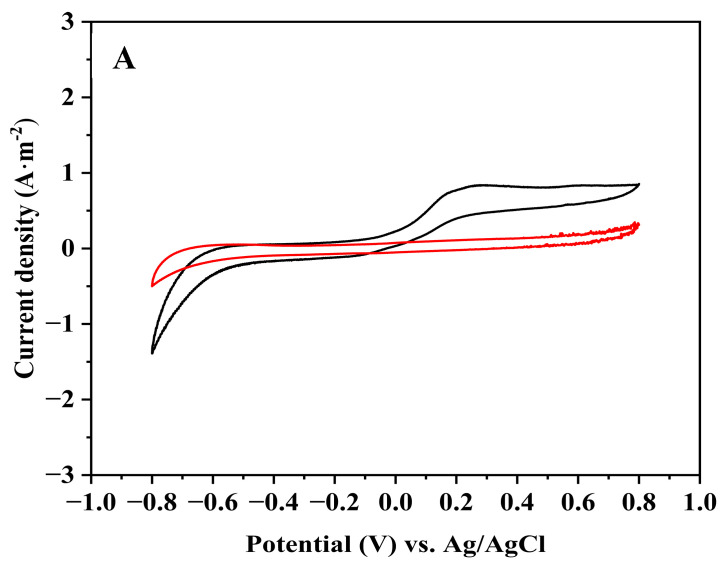

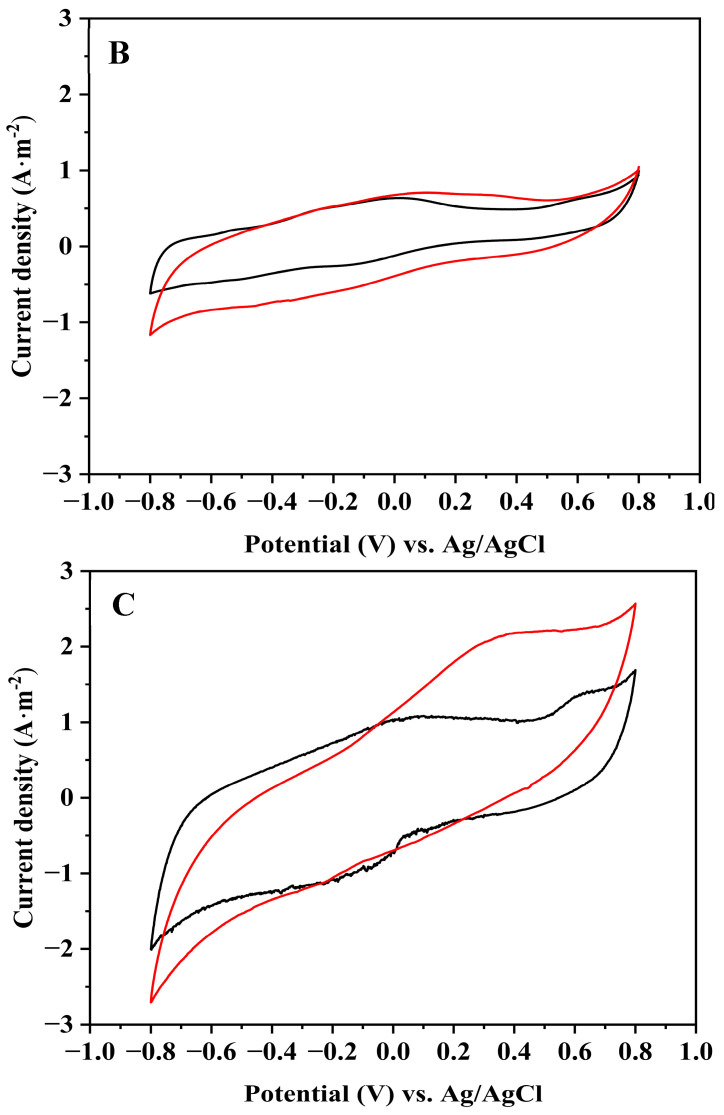
CV of MECs based on the bare anode (**A**), a dialysis-bag anode (**B**), and a graphite-dialysis-bag anode (**C**). MECs were supplied with *Geobacter* medium (black), and artificial wastewater (red). Measurements were performed on the 20th day of the MEC operation.

A set of single-chamber MECs was constructed, based on a carbon cloth anode encapsulated in a dialysis bag including 0.25 g of graphite (designated as MEC based on a graphite-dialysis-bag anode), and a platinum-coated carbon cloth cathode. A similar set was constructed without graphite (designated as MEC based on a dialysis-bag anode), and an MEC set with a bare anode; these two sets were used as controls. Each configuration set included eight replicates. The MECs which were based on an anode with a dialysis bag (with or without graphite) were inoculated with 3 mL of *G. sulfurreducens* directly into the dialysis bag. For the MECs with the bare anode, the inoculation of the *G. sulfurreducens* (3 mL) was injected into the whole volume of the MEC medium. All the MECs were fed with *Geobacter* medium for 14 days. After this period, the feeding of half of the MECs (that is, four replicates from each of the three configurations) continued with *Geobacter* medium, and the other MECs were fed with artificial wastewater for another 14 days. All the MECs’ content medium was replaced once a week, and twice a week were fed with acetate. Cyclic voltammogram (CV, three cycles) and LSV measurements were performed at a potential range of −0.8 to 0.8 V and −0.5 to 0.8 V, respectively, with a scan rate of 5 mV·s^−1^ vs. Ag/AgCl (3.0 M KCl). The CV results on the 20th day of the three MEC configurations which were fed with *Geobacter* medium, or artificial wastewater, are shown in Figure 2. Areal capacitance normalized to the area of active materials was calculated based on Equation (4), shown in Table 1. And LSV results are shown in Table 2.

As shown in Figure 2A–C, the MEC utilizing the graphite-dialysis-bag anode which was fed with *Geobacter* medium and artificial wastewater had the highest electroactivity. When the MECs were fed with *Geobacter* medium, the areal capacitance of the MEC which was based on the graphite-dialysis-bag anode, dialysis-bag anode, and the bare anode was 117.75, 56.25, and 26.25 F·m^−2^, respectively. When the MECs were fed with artificial wastewater, it was 134.13, 82.50, and 13.75 F·m^−2^, respectively (Table 1).

Moreover, LSV analysis (Table 2) of MECs that were fed with *Geobacter* medium and were based on a graphite-dialysis-bag anode, and those based on a dialysis-bag anode, led to 1.29 ± 0.76 and 0.81 ± 0.05 A·m^−2^ at 0.8 V, respectively. The MEC utilizing the bare anode led to only 0.53 A·m^−2^ (2.4-fold less than the graphite-dialysis-bag anode). The same phenomenon was observed when the MECs were fed with artificial wastewater: the highest current density of 2.73 ± 0.49 A·m^−2^ at 0.8 V was observed for the graphite-dialysis-bag anode. The dialysis-bag anode led to 1.73 ± 0.97 A·m^−2^ at 0.8 V. Interestingly, the bare anode, when fed with wastewater, led to only 0.33 ± 0.28 A·m^−2^ (8-fold less than the graphite-dialysis-bag anode).

### 3.2. Electrochemical Impedance Spectroscopy (EIS)

EIS was performed at the beginning of the MECs’ operation with abiotic MECs (before *G. sulfurreducens* inoculation), and again during the experiment. The MECs were connected to the potentiostat in a three-electrode configuration, under an applied constant potential. Impedance was examined with 10^5^–10^−1^ Hz frequencies and an amplitude of 10 mV. The results were fitted with the ZSimpWin (ZSimpWin 3.21) application.

**Figure 3:**
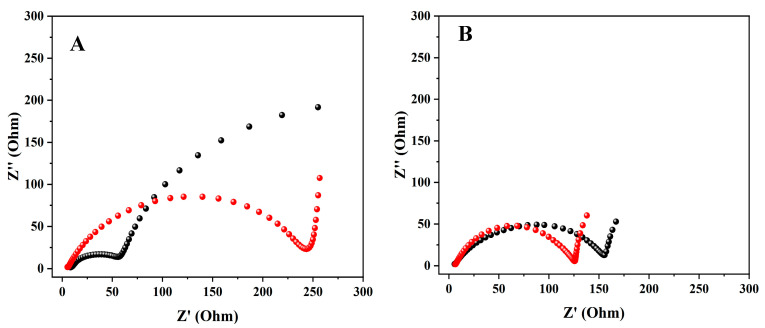

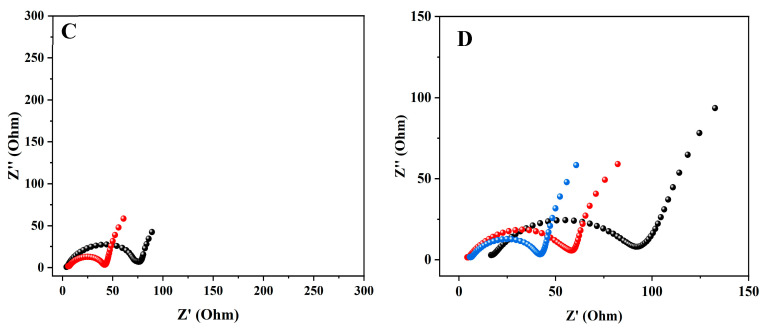
EIS of MECs (on the 20th day) based on a bare anode (**A**); a dialysis-bag anode (**B**); and a graphite-dialysis-bag anode (**C**). MECs were supplied with *Geobacter* medium (black) and artificial wastewater (red); EIS of the MEC based on a graphite-dialysis-bag anode fed with artificial wastewater before inoculation on zero-day (black), on the 21st day (red), and on the 28th day (blue) (**D**).

EIS analysis (Figure 3A–C) showed that when the MECs were fed with artificial wastewater, the resistance of MECs with the graphite-dialysis-bag anode was 35 Ω, and with a dialysis-bag anode it was 120 Ω, compared to the same MECs which were fed with the *Geobacter* medium (71 Ω and 148 Ω, respectively). However, the MEC based on the bare anode showed the opposite phenomenon. When it was fed with artificial wastewater the resistance was higher than when it was fed with *Geobacter* medium: 238 Ω and 49 Ω, respectively.

The EIS of the MEC that was based on the graphite-dialysis-bag anode and supplied with artificial wastewater showed that before bacterial inoculation at time zero, the resistance was the highest (75 Ω). During bacterial anode development, the resistance was decreased; on day 21, it was 54 Ω, and on the 28th day, it was only 35 Ω.

### 3.3. Hydrogen Production Rate in the MECs

**Figure 4:**
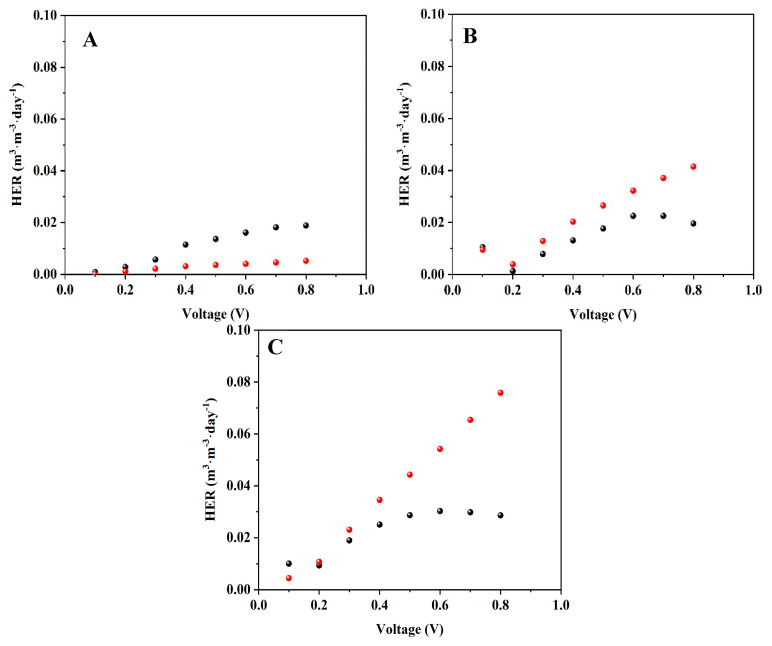
Hydrogen production rate as a function of applied voltages of MECs utilizing the bare anode (**A**), dialysis-bag anode (**B**), and graphite-dialysis-bag anode (**C**). When the MECs were fed with *Geobacter* medium (black), and artificial wastewater (red).

LSV for reduction currents was performed on the 20th day of operation in a two-electrode configuration, at a potential range of 0 to −0.8 V, and a scan rate of 5 mV·s^−1^. The currents were calculated to yield HER using Equation (1). The results are shown in Figure 4.

The highest HER was obtained by the MEC which utilized the graphite-dialysis-bag anode and was supplied with artificial wastewater. It is important to note that in this MEC, there was a linear correlation with the HER and the increase in the applied voltage. For example, the HER was 2.3 × 10^−2^, 5.4 × 10^−2^, and 7.6 × 10^−2^ m^3^·m^−3^·day^−1^ for 0.3, 0.6, and 0.8 applied voltages, respectively.

In addition, in all the applied voltages, the MECs with the dialysis-bag anode with or without the graphite, which were fed with the artificial wastewater, showed higher HER (7.6 × 10^−2^ and 4.2 × 10^−2^ m^3^·m^−3^·day^−1^, respectively) than when they were fed with *Geobaceter* medium (2.9 × 10^−2^ and 2.0 × 10^−2^ m^3^·m^−3^·day^−1^ at 0.8 V, respectively).

This phenomenon is opposed to the HER obtained by the MEC with the bare anode, where the HER was higher when it was fed with a *Geobaceter* medium compared to wastewater. At 0.8 V the HER was 1.9 × 10^−2^ m^3^·m^−3^·day^−1^ for the MEC which was fed with *Geobaceter* medium, and only 5.1 × 10^−3^ m^3^·m^−3^·day^−1^ when it was fed with artificial wastewater. We assume that when the MEC with the bare anode was fed with the artificial wastewater, non-exoelectrogenic bacteria interrupted the electron transfer by the exoelectrogenic bacteria to the anode material.

In contrast, when the anode was encapsulated by the dialysis bag, the dialysis bag served as a barrier and inhibited the invasion and settlement of non-exoelectrogenic bacteria on the carbon cloth anode.

### 3.4. The Contribution of the Planktonic Bacteria to the Overall Evolved Currents of the MECs

The contribution of the planktonic bacteria to the overall obtained currents in the different MEC configurations was examined at the end of the MEC operation (28th day). For this examination, the bacterial anodes from all the MECs were replaced with sterile ones. In addition, the oxidation currents of the complete MEC configuration were examined (Table 3) by LSV in a three-electrode configuration, at a potential range of 0.2 to 0.8 V, and at a scan rate of 5 mV·s^−1^ vs. Ag/AgCl (3.0 M KCl).

As shown in Table 3, the increase of currents in all the different complete MEC configurations and the currents evolved from the planktonic bacteria were correlated with the increase of the applied voltages. In addition, in all MECs, at an applied voltage of 0.8 V, the highest currents were obtained when the MECs were fed with *Geobacter* medium, compared to when fed with artificial wastewater. For the MECs based on the graphite-dialysis-bag anode, the values were 1.54 ± 0.24 and 2.62 ± 0.29 A·m^−2^, respectively. For the MECs based on the dialysis-bag anode and bare anode, the current was 0.94 ± 0.42, 1.28 ± 0.22 A·m^−2^; and 0.53 ± 0.36, 0.33 ± 0.16 A·m^−2^, respectively. The planktonic bacteria contributed about a third to a seventh of the currents obtained by the complete MEC in all the configurations. The abiotic MECs showed negligible electrochemical activity. For example, at an applied voltage of 0.8 V, MECs based on the graphite-dialysis-bag anode and fed with *Geobacter* medium led to only 0.62 ± 0.11 A·m^−2^. Similar results were obtained by the other MEC configurations in the abiotic environment.

Several studies have modified the bacterial anode in an attempt to preserve the exoelectrogenic bacteria in the biofilm on the carbon cloth anode or to increase the anode surface area. In our previous study, we used a single-chamber MEC with a carbon cloth anode and *G. Sulfurreducens* encapsulated in a gelatine capsule, which was then coated with water-insoluble cellulose acetate; and a carbon cloth cathode coated with Pt. The results showed that under an applied voltage of 0.6 V, when the MEC was fed with artificial wastewater, the current density and the calculated HER were 1.7 A·m^−2^ and 2.7 × 10^−2^ m^3^·m^−3^·day^−1^, respectively [21]. In comparison, our current study showed that the graphite-dialysis-bag anode led to a current density and HER of 7.9 × 10^−1^ A·m^−2^ and 5.4 × 10^−2^ m^3^·m^−3^·day^−1^, respectively. Yong et al. (2013) presented a new technique for immobilizing *Shewanella oneidensis* in an MFC (dual chamber separated by a Nafion membrane). The anode chamber was constructed of graphite-alginate granules, with carbon cloth which was used for both anodic and cathode electrodes. Nyquist plots of the MFC with the graphite-alginate-granule anode showed a charge transfer resistance of 1600 Ω [20]. In our current study, EIS analysis (Figure 3C) showed charge transfer resistance of 35 Ω and 71 Ω in the MEC which was based on the graphite-dialysis-bag anode, when fed with artificial wastewater and *Geobacter* medium, respectively. Yasri et al. (2017) investigated the use of granular activated carbon (GAC) as a high-surface area 3-dimensional (3D) anode in MECs. When the electrodes were powered at a voltage of 1.0 V, the MEC which was based on Fe_3_O_4_-GAC led to a current of 29.8 A·m^−3^, an HER of 0.36 m^3^·m^−3^·d^−1^, and EIS (Rct) of 204 Ω [24]. Rozenfeld et al. (2021) focused on a semi-single-chamber MEC based on an anode made of carbon cloth treated with plasma and encapsulated in a dialysis bag (50 kDa), and carbon cloth coated with Pt as a cathode. The encapsulated anode was inoculated with a small volume of *G. sulfurreducens* entered directly into the dialysis bag. The HER obtained was 0.49 m^3^·m^−3^·d^−1^. When the MEC was fed with wastewater, the current density was 12.40 A·m^−3^, compared to feeding with acetate (15.70 A·m^−3^), under an applied voltage of 0.6 V vs. Ag/AgCl [22]. Gandu et al. (2020) examined a single-chamber MEC based on *G. sulfurreducens*, which was encapsulated on a carbon cloth anode using alginate and chitosan (AC-1), and fed with wastewater. The AC-1 anode led to a current density of 5.96 A·m^−2^ and a HER rate of 0.56 m^3^·m^−3^·d^−1^ (at 0.5 V) [23]. Rani et al. (2021) examined MECs fed with dairy industry wastewater. A carbon cloth anode and a graphite sheet cathode were ingrained with Fe_3_O_4_ nanoparticles (FNP); MECs with uncoated electrodes were used as the control. The current density according to CV for the control FNP-coated electrode was 0.66 A·m^−2^. EIS showed the bulk resistivity of the FNP-coated electrodes as 400 Ω, which was 10 times higher than that of the MEC utilizing the graphite-dialysis-bag anode in our current study (35 Ω) [25]. Zikmund et al. (2018) reported on a two-chamber MEC separated by an AEM (anion-exchange membrane). A cylinder-shaped graphite-fiber brush was used as the anode, and discs of stainless-steel mesh containing a platinum catalyst was used as the cathode. The results showed an average current density and HER of 4.2 A·m^−2^ and 0.38 m^3^·m^−3^·d^−1^, respectively [26].

### 3.5. COD Removal and Coulombic Hydrogen Recovery in the MECs

During the period between the 17th and 25th day, all MECs were examined for COD removal. The initial concentrations were measured right after the replacement of the *Geobacter* medium or artificial wastewater on the 17th day. The analysis was conducted using a high-range kit tube. All samples were filtered, appropriately diluted based on their concentration, gently mixed, and processed in the COD reactor. The results were subsequently analyzed using spectrophotometry. Coulombic hydrogen recovery, the ratio of the actual charge involved in the electrochemical reaction to the theoretically calculated charge required for the complete reduction of protons to hydrogen, was calculated using Equation (3).

**Figure 5:**
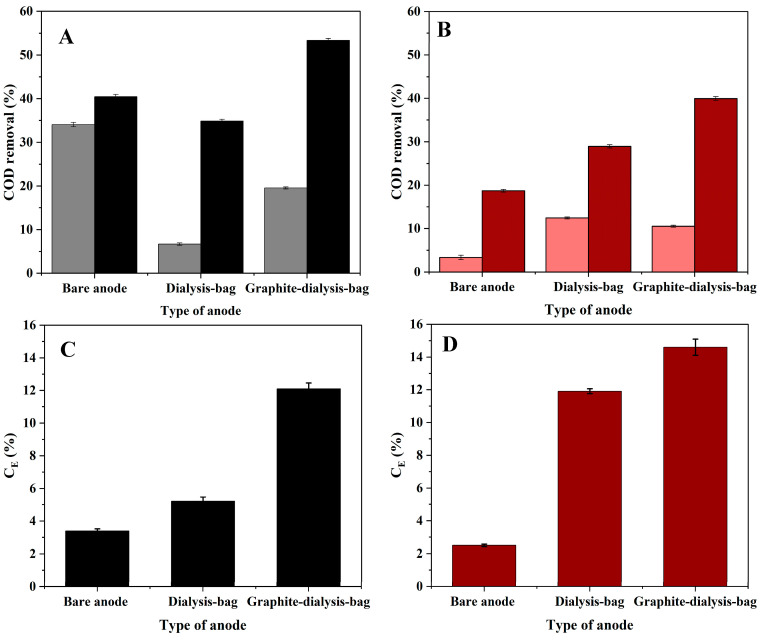
COD removal (%) (**A**,**B**) on 20th (bright columns) and 25th (dark columns) day. Coulombic hydrogen recovery (**C**,**D**) in MECs based on different types of anodes (bare anode, dialysis-bag anode, and graphite-dialysis-bag anode), which were supplied with *Geobacter* medium (black) and artificial wastewater (red).

The results in Figure 5 show COD removal (%) three and eight days after *Geobacter* medium (Figure 5A) or artificial wastewater replacement (Figure 5B). In the systems that were fed with *Geobacter* medium, the percentage of COD removal was higher than in systems fed with artificial wastewater. For example, in the MEC based on the graphite-dialysis-bag anode, the COD removal on the 25th day was 53% when fed with *Geobacter* medium. When fed with artificial wastewater, it was only 40%. In addition, the COD removal was higher on the 25th day, compared to the 20th day, in all the MEC configurations. In the MEC based on the graphite-dialysis-bag anode when fed with artificial wastewater, it was 11% and 40%, respectively.

The coulombic hydrogen recovery (Figure 5) in MECs fed with *Geobacter* medium (C) and artificial wastewater (D) showed that the highest values were for the MEC based on a graphite-dialysis-bag anode (12% and 15%, respectively). For the MEC based on a dialysis-bag anode, it was 5% and 12%, respectively; and for the MEC utilizing the bare anode, it was about 3% in both cases.

Hirsch et al. (2023) presented an MFC system with a dual-glass chamber separated by a proton-exchange membrane (200 mL in both anode and cathode chambers). The cathode was carbon cloth with 0.5 mg·cm^−2^ Pt (10 cm^2^). A mixture of kaolin and bacteria, with or without activated carbon immobilized on a carbon cloth, were used as anodes (kaolin-AC and kaolin, respectively). After 16 days, the MFCs were fed with byre (cowshed) wastewater (COD of 800 mg·L^−1^) for another 23 days. The highest coulombic efficiency of 16% was produced by the MFC based on the kaolin-AC anode, with COD removal of 58% [18]. In a similar system, graphite nanoparticles were used instead of activated carbon. The anodes were composed of a mixture of kaolin (12.5 g·L^−1^) and three different concentrations of graphite nanoparticles (0.25, 1.25, and 2.5 g·L^−1^). The kaolin–graphite (1.25 g·L^−1^) anode exhibited the highest coulombic efficiency (21%), compared with the kaolin-only (17%) and the bare (14%) anodes [27]. Gandu et al. (2020) studied MECs based on an alginate–chitosan (1.0 OD_590_ nm) anode; in this MEC, the COD removal was 75% [23]. Zikmund et al. (2018) used a graphite fiber brush anode, where the coulombic efficiency was 74% [26]. Yasri et al. (2017) examined coulombic efficiency and COD removal in an MEC based on Fe_3_O_4_ as granular-activated carbon, and they obtained results of 96.7% and 57%, respectively [24]. Yong et al. (2013) showed that the coulombic efficiency of an MFC utilizing an anode composed of *Shewanella oneidensis* immobilized in graphite–alginate granules was higher (1.79-fold) than that of an MFC with the cells in suspension [20]. Luo et al. (2016) presented an MFC (28 mL) with a cylindrical shape using a carbon cloth anode (diameter of 1 cm and height of 2 cm) and a plastic mesh as a separator, along with a Pt-catalyst air-cathode. Municipal wastewater treatment plant cells were used as a source of inoculum, immobilized to the anode by agar gel, and 10 g·L^−1^ sodium acetate was used as a carbon source. The COD removal reached about 90%, and the coulombic efficiency was 10.7% [28].

### 3.6. Bacterial Biofilm Anode and Planktonic Bacteria Viability

At the end of our experiment (day 28), the viability of the bacterial biofilm on the carbon cloth anodes was examined, based on tetrazolium salt reduction by the bacterial hydrogenase (MTT analysis). The viability of the planktonic bacteria in the medium was also measured using MTT analysis. In the MECs based on the dialysis-bag anode, with or without graphite, the MTT analysis was measured from the medium inside the dialysis bag. The viability of the planktonic bacteria in the MEC based on a bare anode was tested from a whole-volume sample. The samples were centrifuged, and the sediment was washed with PBS 3 times before the MTT analysis.

**Figure 6:**
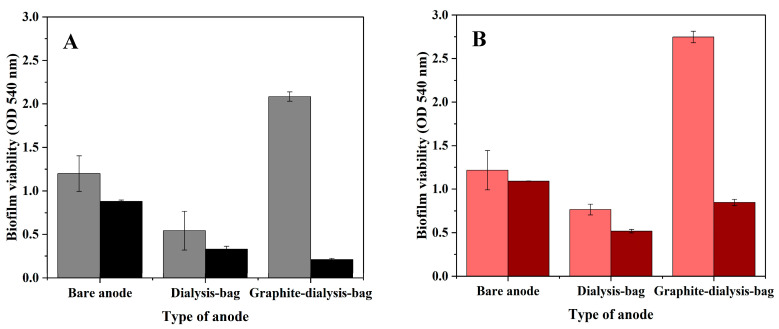
MTT analysis of the bacterial biofilm anode (bright columns) and planktonic bacteria (dark columns) in the MECs based on the bare anode, dialysis-bag anode, and graphite-dialysis-bag anode fed with *Geobacter* medium (**A**), and artificial wastewater (**B**), on day 28 of MEC operation.

The absorbance intensity of the purple solution was examined using a spectrophotometer at 540 nm (Figure 6).

The results in Figure 6 showed that the viability of the bacterial biofilm anode was higher than the viability of the planktonic bacteria. In MECs fed with artificial wastewater (B), the viability of the biofilm anode in the MEC based on a graphite-dialysis-bag anode was 2.75 ± 0.07 OD_540_ nm, and for the planktonic bacteria it was only 0.85 ± 0.04 OD_540_ nm. In addition, in the MEC utilizing the graphite-dialysis-bag anode, the viability of the planktonic bacteria and the bacterial anode were higher when fed with artificial wastewater (B) compared to *Geobacter* medium (A): 0.85 ± 0.04 vs. 2.75 ± 0.07, and 0.21 ± 0.01 vs. 2.08 ± 0.05, respectively. It can thus be seen that graphite nanoparticles increased the viability of the bacterial anode. We assume that a portion of the nanoparticles attached to the biofilm enabled electron transfer from the bacteria to the anode material, which enhanced the bacterial viability.

### 3.7. Microbial Diversity of the Biofilm on the Carbon Cloth Anodes

**Figure 7:**
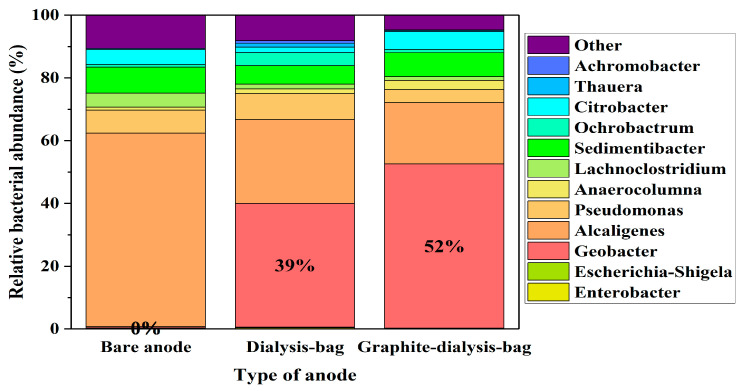
Relative bacterial distribution at the genus level on the biofilm anodes in MECs based on the bare anode, dialysis-bag anode, and graphite-dialysis-bag anode, which were supplied with artificial wastewater.

The microbial diversity of the MECs utilizing the bare anode, dialysis-bag anode, and graphite-dialysis-bag anode was assessed based on 16S rRNA after 28 days of the MEC operation (when fed with wastewater). Operational taxonomic unit (OTU) readings were identified and phylogenetically classified. Unidentified species or sequences with relative abundances of <0.5% were collectively categorized as “Others/NA” (Figure 7). The results are based on two replicates of each anode.

*G. sulfurreducens* is an anaerobic bacterium, related to the class of *Deltaproteobacteria*. Its allocation on the MECs based on the bare anode, dialysis-bag anode, and graphite-dialysis-bag anode, when fed with artificial wastewater, were 0.2%, 39%, and 52%, respectively. The low percentage of *G. sulfurreducens* in the MEC based on the bare anode and fed with artificial wastewater can be explained by the presence of other bacteria competing with the exoelectrogenic *G. sulfurreducens*.

In MECs utilizing the dialysis-bag anode, with or without the graphite nanoparticles, the most abundant bacteria were *Geobacter*. This phenomenon was observed by other studies. Luo et al. (2016) presented an MFC based on an anode made of *Geobacter* immobilized by agar gel, where the highest relative abundance of bacteria was *Geobacter* (62%) [28]. Gandu et al. (2020) showed an MEC based on a bacterial anode immobilized with alginate and chitosan, the *G. sulfurreducens’* relative distribution was 92% [23].

*Escherichia* and *Enterobacter,* which were added to the artificial wastewater, were found in negligible amounts, less than 1%. Several bacterial cells that were found in all the bacterial anodes had probably contaminated the anode before the construction of the MEC. We assume that the contamination evolved from non-efficient sterilization methods of the anode material, including the dialysis bag.

The abundance of *Alcaligenes* on the bare bacterial anode was 62% when the MEC was fed with artificial wastewater (Figure 7). *Alcaligenes* are aerobic, and the facultative anaerobic bacteria belonging to the *Alcaligenaceae* family are widely distributed in various environments, including soil, water, and clinical settings. Certain strains of *Alcaligenes* have been studied for their potential applications in BES systems, due to their ability to degrade various organic compounds, their tolerance to environmental stresses, and their electroactive possibilities. Yu et al. (2018) showed that *Alcaligenes faecalis* catalyzed an outward extracellular electron transfer and generated electricity at an applied voltage of 0.3 V vs. SHE [29]. In Wang et al. (2015), the *Alcaligenes* sp. was reported as the acceptor of the cathodic electrons for nitrate reduction [30]. Li et al. (2024) concluded that the strain *A. faecalis* ZS-1, which relies on electrogenesis, employs an internal electron transfer pathway to convert CO_2_ by receiving electrons from the cathodic electrode [31]. In addition, *Pseudomonas* species were observed in the different MEC bacterial anodes. As mentioned in several studies, *Pseudomonas* strains are prevalent within the microbial community of MFCs and are recognized for releasing electron mediators like pyocyanin and phenazine, possessing a redox potential [32,33,34].

In summary, encapsulating the anode in a dialysis bag that served as a physical barrier preserved the most abundant amount of the exoelectrogenic *G. sulfurreducens.* Most of the contaminated bacteria evolved from unsuitable methods of sterilization of the anode components, including the dialysis bag. In addition, a dialysis bag anode in a wastewater plant needs to resist shear and friction forces. Thus, in a sustainable MEC, the dialysis bag should be replaced with a stable porous material that will serve as the physical barrier to preserve the exoelectrogenic bacteria.

## 4. Conclusions

In this study, the bacterial anode was encapsulated by a dialysis bag including suspended graphite particles to improve current transfer from the bacteria to the anode material. An anode encapsulated in a dialysis bag without graphite particles and a bare anode were used as controls. It was observed that the MEC utilizing the graphite-dialysis-bag anode which was fed with *Geobacter* medium and artificial wastewater had the highest electroactivity. When the MECs were fed with *Geobacter* medium, the areal capacitance of the MECs based on the graphite-dialysis-bag anode, dialysis-bag anode, and bare anode was 117.75, 56.25, and 26.25 F·m^−2^, respectively. LSV analysis showed that MECs based on the graphite-dialysis-bag and dialysis-bag anodes led to 1.29 ± 0.76 and 0.81 ± 0.05 A·m^−2^ at 0.8 V, respectively. The MEC utilizing the bare anode led to only 0.53 ± 0.25 A·m^−2^. The graphite-dialysis-bag anode led to the highest HER of 7.6 × 10^−2^ m^3^·m^−3^·day^−1^ at an applied voltage of 0.8 V and the highest COD removal of 53% when fed with *Geobacter* medium. The bacterial distribution on the biofilm anode showed that the graphite-dialysis-bag anode hosted 52% *G. sulfurreducens*, compared with the dialysis-bag anode, 39%, and the bare anode, only 0.2%. The results showed that the addition of the graphite particles increased the bacterial anode electroactivity. In addition, encapsulating the anode in a dialysis bag that served as a physical barrier preserved the most abundant amount of the exoelectrogenic *G. sulfurreducens.* However, a dialysis bag anode in a wastewater plant needs to resist shear and friction forces. Thus, in a sustainable MEC, the dialysis bag should be replaced with a stable porous material which will serve as the physical barrier.

## Figures and Tables

**Table 1:** Summary of areal capacitance.

	Areal Capacitance (F·m^−2^)		
Type of Anode	Bare Anode	Dialysis-Bag Anode	Graphite-Dialysis-Bag Anode
*Geobacter* medium	26.25	56.25	117.75
Artificial wastewater	13.75	82.50	134.13

**Table 2:** Summary of LSV current densities.

	Current Density at an Applied Voltage of 0.8 V (A·m^−2^)		
Type of Anode	Bare Anode	Dialysis-Bag Anode	Graphite-Dialysis-Bag Anode
*Geobacter* medium	0.53 ± 0.25	0.81 ± 0.05	1.29 ± 0.76
Artificial wastewater	0.33 ± 0.28	1.73 ± 0.97	2.73 ± 0.49

**Table 3:** LSV analysis of the planktonic bacteria and the complete MECs in the different configurations.

Carbon Source	Electrode	Current Source	Current Density (A·m^−2^)			
			0.2 V	0.4 V	0.6 V	0.8 V
*Geobacter* medium	Bare anode	Complete MEC	0.39 ± 0.11	0.42 ± 0.29	0.48 ± 0.32	0.53 ± 0.36
		Planktonic bacteria	0.03 ± 0.01	0.04 ± 0.01	0.06 ± 0.01	0.17 ± 0.04
	Dialysis-bag anode	Complete MEC	0.42 ± 0.30	0.35 ± 0.22	0.45 ± 0.33	0.94 ± 0.42
		Planktonic bacteria	0.02 ± 0.01	0.02 ± 0.01	0.05 ± 0.03	0.13 ± 0.04
	Graphite-dialysis-bag anode	Complete MEC	0.58 ± 0.24	0.81 ± 0.05	1.05 ± 0.02	1.54 ± 0.24
		Planktonic bacteria	0.16 ± 0.08	0.20 ± 0.08	0.35 ± 0.10	0.62 ± 0.11
Artificial wastewater	Bare anode	Complete MEC	0.17 ± 0.06	0.21 ± 0.09	0.23 ± 0.09	0.33 ± 0.16
		Planktonic bacteria	0.03 ± 0.01	0.06 ± 0.02	0.10 ± 0.03	0.13 ± 0.05
	Dialysis-bag anode	Complete MEC	0.96 ± 0.50	1.08 ± 0.14	1.17 ± 0.24	1.28 ± 0.22
		Planktonic bacteria	0.03 ± 0.01	0.07 ± 0.01	0.14 ± 0.11	0.37 ± 0.19
	Graphite-dialysis-bag anode	Complete MEC	1.31 ± 0.24	1.59 ± 0.10	1.81 ± 0.02	2.62 ± 0.29
		Planktonic bacteria	0.11 ± 0.03	0.18 ± 0.05	0.25 ± 0.06	0.65 ± 0.06

## Data Availability

The original contributions presented in the study are included in the article, further inquiries can be directed to the corresponding author.
